# High Removal Rates and Atomically Smooth Surfaces Are Achieved on Silicon Wafers Using the New SiO_2_@ZrO_2_ Core–Shell Abrasive

**DOI:** 10.3390/nano16171064

**Published:** 2026-08-26

**Authors:** Maokui Wang, Weilong He, Kai Feng, Rui Ye, Yunci Wang, Weiyi Guan, Facheng Qiu, Xing Fan, Renlong Liu

**Affiliations:** 1 College of Chemistry and Chemical Engineering, Chongqing University, Chongqing 400044, China; 2 Hubei Sinophorus Electronic Materials Co., Ltd., Yichang 443007, China; 3 College of Chemistry and Chemical Engineering, Chongqing University of Technology, Chongqing 400054, China

**Keywords:** chemical mechanical polishing (CMP), SiO_2_@ZrO_2_, composite abrasives, atomic-level surface roughness, silicon wafer planarization

## Abstract

The relentless miniaturization of integrated circuits demands chemical mechanical polishing (CMP) technologies that achieve both high material removal rates (MRR) and atomic-scale surface quality. Herein, we synthesize sub-100 nm SiO_2_@ZrO_2_ core–shell composite abrasives (amorphous SiO_2_ core ~70 nm, tetragonal ZrO_2_ shell ~6 nm) via a facile sol–gel method. Electron microscopy and X-ray photoelectron spectroscopy strongly indicated uniform core–shell architecture and Si–O–Zr covalent bonding essential for stable coating. Polishing tests show that the abrasives deliver an MRR of 353.54 nm/min—approximately 2.8 times that of pure SiO_2_—and reduce surface roughness to Ra = 0.105 ± 0.015 nm, approaching atomic-scale planarization. The superior performance stems from the rigid ZrO_2_ shell, which suppresses elastic deformation and preserves spherical contact morphology. This mechanical effect simultaneously increases shear stress by reducing contact area and minimizes scratches by limiting indentation depth. Overall, this work offers a simple, controllable strategy for designing high-efficiency CMP abrasives and demonstrates the considerable potential of SiO_2_@ZrO_2_ core–shell materials for damage-free, atomic-scale surface finishing.

## 1. Introduction

Chemical mechanical polishing (CMP), as a critical technology for achieving global planarization of wafers in integrated circuit manufacturing, directly determines the interconnect performance and final yield of devices [1,2,3]. As the feature size of chips continuously shrinks to nanometer nodes in the post-Moore era, the requirements for surface planarity and defect control of silicon wafers have reached the atomic scale [4]. Particularly in the planarization processes of shallow trench isolation (STI) and interlayer dielectric (ILD), the polishing quality of silicon dioxide (SiO_2_) films directly affects the integrity of the gate oxide layer and the control of parasitic capacitance [5,6,7,8]. Consequently, developing a novel polishing slurry capable of simultaneously achieving a high material removal rate (MRR) and a near-atomically damage-free surface has become an urgent bottleneck problem in advanced semiconductor manufacturing.

Currently, traditional pure SiO_2_ abrasives are widely used owing to their excellent dispersibility and chemical stability [9,10]. However, silicon dioxide (SiO_2_) possesses a Mohs hardness of 7 and a relatively low elastic modulus of approximately 70 GPa [11], making it highly susceptible to elastic deformation under polishing pressure. This deformation alters the contact geometry between the abrasive particles and the wafer surface, inducing a transition from rolling friction to sliding friction and exacerbating the “plowing effect.” Consequently, this not only constrains the enhancement of material removal efficiency but also readily introduces scratches and subsurface damage to the substrate [12]. To overcome this limitation, researchers have attempted to composite SiO_2_ with highly active metal oxides such as CeO_2_, constructing SiO_2_@CeO_2_ core–shell abrasive systems [12,13,14]. The removal mechanism of this system is primarily attributed to the abundant Ce^3+^/Ce^4+^ redox pairs on the CeO_2_ surface, which promote material removal through the formation of Ce–O–Si bonds, and indeed elevate the MRR to a certain extent. However, CeO_2_ has a Mohs hardness comparable to that of SiO_2_ [15], making the abrasive prone to plastic deformation at the contact interface and thereby exacerbating the “plowing effect.” As a result, the surface roughness (Rq) after polishing with SiO_2_@CeO_2_ systems typically remains above 0.4 nm, failing to meet the requirements for near atomic-scale smoothness [14]. Moreover, commercial CeO_2_ abrasives often possess irregular morphologies and broad particle size distributions, which readily introduce scratch defects.

In view of the surface damage caused by hard abrasives, recent research interest has shifted toward flexible polishing strategies, i.e., constructing mSiO_2_@CeO_2_ composite abrasives with mesoporous silica (m SiO_2_) as the core [16,17,18,19]. Unlike solid SiO_2_, m SiO_2_ possesses an ordered pore structure and a substantially reduced Young’s modulus, enabling it to act as a “spring” during polishing contact, absorbing impact energy through an elastic cushioning effect. This effectively suppresses scratches and reduces the surface roughness to approximately 0.2 nm [19]. However, such a strategy of “overcoming hardness with softness” has come at a huge expense in efficiency: the introduction of the mesoporous structure drastically weakens the overall mechanical strength of the abrasive, causing the material removal rate to drop sharply to below 100 nm/min. For instance, the reported material removal rates are 64 nm/min for abrasives with mesoporous silica cores [20] and 85 nm/min for those with dendritic mesoporous silica cores [21], which severely limits its practical application in mass production. In summary, the design of existing CMP abrasives has fallen into the dilemma that “hard abrasives cause damage, soft abrasives sacrifice rate”.

Zirconia (ZrO_2_) emerges as a compelling alternative to resolve this dilemma. First, ZrO_2_ possesses a substantially higher hardness (Mohs hardness 8.5) [22] than both SiO_2_ and CeO_2_, enabling it to resist elastic deformation and maintain spherical contact morphology under polishing pressure. Second, ZrO_2_ exhibits excellent chemical stability across a wide pH range and does not aggressively decompose H_2_O_2_, ensuring robust performance in CMP slurry environments. Third, zirconium precursors are commercially available at relatively low cost, offering good scalability for industrial production. Most importantly, the formation of Zr–O–Si chemical bonds between ZrO_2_ and SiO_2_ surfaces during CMP has been experimentally verified by Dai et al. [23], indicating that ZrO_2_ can participate in the chemical removal process analogous to CeO_2_. Theoretically, if rigid ZrO_2_ serves as the shell material, its high modulus would resist elastic deformation and maintain spherical abrasive morphology, which is expected to significantly reduce indentation depth under the same applied pressure, thereby avoiding brittle removal and scratch generation [24].

Despite these advantages, systematic research on the controlled preparation of sub-100 nm SiO_2_@ZrO_2_ core–shell abrasives and their structure–property relationship in silicon wafer CMP has rarely been reported. To the best of our knowledge, achieving an amorphous SiO_2_ core with a crystalline tetragonal ZrO_2_ shell below 100 nm in size and demonstrating its CMP performance remains an unexplored frontier.

Based on this, the present work proposes a “rigid constraint” strategy and successfully designs and synthesizes sub-100 nm SiO_2_@ZrO_2_ core–shell composite abrasives via a sol–gel method. By adjusting the zirconium precursor concentration and aging time, the growth of the tetragonal ZrO_2_ shell was precisely controlled, and X ray photoelectron spectroscopy confirmed the presence of Si–O–Zr covalent bonds, thereby ensuring structural stability. Systematic polishing experiments demonstrate that this abrasive breaks the conventional “efficiency–quality” trade off, simultaneously delivering a high material removal rate of 353.54 nm/min (approximately 2.8 times that of pure SiO_2_) and a near atomically smooth surface with Ra = 0.105 ± 0.015 nm. This study further elucidates the modulation mechanism of the rigid ZrO_2_ shell on the contact mechanical behavior, providing a new perspective for the design of next-generation high-performance CMP abrasives.

## 2. Materials and Methods

### 2.1. Materials and Reagents

Zirconium n-butoxide (ZrO_4_C_16_H_36_, 80%), γ-polyglutamic acid (γ-PGA, LR) and sodium hydroxide solution (NaOH, 30%) were supplied by Macklin Biochemical Technology Co., Ltd. (Shanghai, China). Ammonium hydroxide (NH_3_·H_2_O, 25–28%) and hydrogen peroxide (H_2_O_2_, 30%) were purchased from Chongqing Wansheng Chuandong Chemical Co., Ltd. (Chongqing, China). Tetraethyl orthosilicate (TEOS, AR) and anhydrous ethanol (C_2_H_5_OH, AR) were purchased from Chengdu Kelong Chemical Co., Ltd. (Chongqing, China). All reagents were used as received without further purification. Deionized water was used throughout all experiments.

### 2.2. Preparation of Spherical SiO_2_

Monodisperse silica spheres were prepared via a modified Stöber method [25]. In a typical synthesis, 180 mL of anhydrous ethanol, 9 mL of ammonium hydroxide, and 4.5 mL of TEOS were thoroughly mixed and stirred at room temperature for 16 h (600 r/min). The obtained product was dried at 100 °C to yield nano-spherical silica.

### 2.3. Preparation of SiO_2_@ZrO_2_

**Figure 1:**
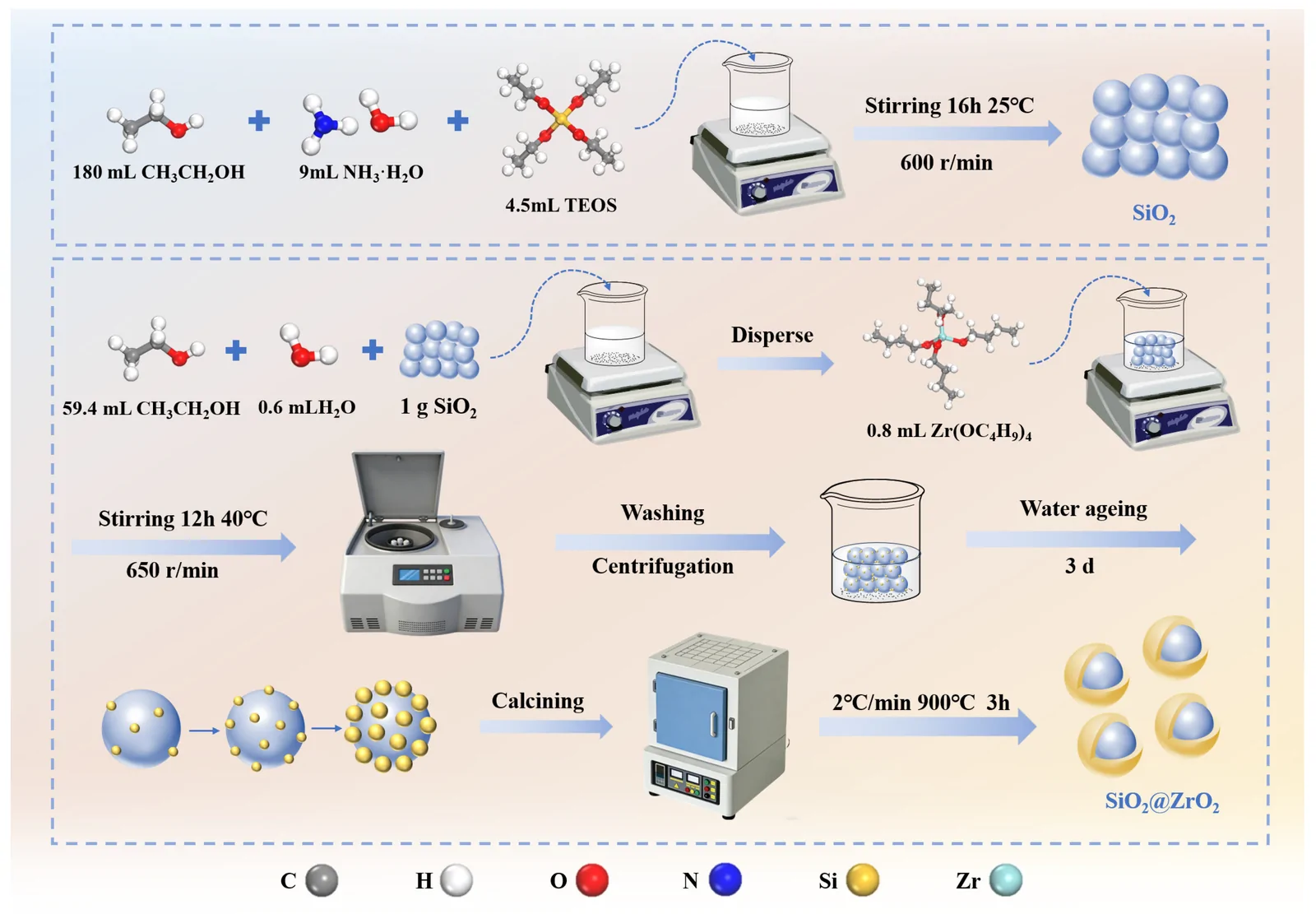
Flowchart of the preparation process of SiO_2_@ZrO_2_.

1 g of the as-prepared silica particles was dispersed in a mixed solution of 59.4 mL of anhydrous ethanol and 0.6 mL of deionized water, followed by the addition of 0.8 mL of ZrO_4_C_16_H_36_. The reaction vessel was placed in a water bath at 40 °C and stirred for 12 h (650 r/min). The resulting product was then repeatedly centrifuged and washed with deionized water, re-dispersed in water, and aged for 3 days. After centrifugation, the sample was dried at 90 °C and finally, the material was prepared by heating it in a muffle furnace in an air atmosphere at a heating rate of 2 °C/min to 900 °C and then maintaining the temperature for 3 h, resulting in a SiO_2_@ZrO_2_ core–shell composite material. The schematic diagram is illustrated in Figure 1.

### 2.4. Characterization Methods

The size, morphology, and structure of the samples were characterized using scanning electron microscopy (SEM, SU8600, Hitachi, Tokyo, Japan) and transmission electron microscopy (TEM, Talos F200S, Thermo Fisher Scientific Co., Ltd., Brno, Czech Republic) equipped with energy-dispersive X-ray spectroscopy (EDS) and selected-area electron diffraction (SAED). X-ray diffraction (XRD, Panalytical, Almelo, The Netherlands) patterns were recorded over a 2θ range of 5–90° to analyze the crystalline phases. Fourier transform infrared spectrometer (FT-IR, Nicolet iS50, Thermo Fisher Scientific Co., Ltd., Waltham, MA, USA) was used to obtain the infrared spectrum in the range of 4000–400 cm^−1^. The chemical states of the surface elements were examined by X-ray photoelectron spectroscopy (XPS, K-Alpha, Thermo Fisher Scientific Co., Ltd. Waltham, MA, USA). Raman spectroscopy (Raman, inVia Reflex, RENISHAW, Gloucestershire, UK) was employed to further identify the crystalline phase of ZrO_2_. The dispersibility of the polishing solution before and after the addition of the dispersant was characterized using a nanoparticle size and Zeta potential analyzer (DLS and Zeta, NanoBrook Omni, Brookhaven Instruments, Worcestershire, UK).

### 2.5. Polishing Tests and Evaluations

The polishing slurry consisted of abrasive particles (SiO_2_ or SiO_2_@ZrO_2_, 0.5 wt%), an oxidizer (H_2_O_2_, 1 wt%), a dispersant (γ-PGA, 1 wt%), and deionized water, with a total slurry volume of 50 mL; all percentages are given as mass percentages relative to the total slurry. Finally, the pH of the solution was adjusted to 8 with dilute NaOH solution. The components were thoroughly mixed and ultrasonicated to ensure homogeneous dispersion. H_2_O_2_ was selected as the oxidizer due to its environmentally benign nature, as it introduces no metallic impurities and effectively oxidizes the silicon surface to Si–OH species [26]. This surface hydroxylation is crucial for the “chemical tooth” removal mechanism [27]. Meanwhile, γ-PGA is a biodegradable anionic polymer that functions as a high-performance dispersant through the synergistic effects of steric hindrance and electrostatic repulsion. Its inherent biodegradability also makes it an environmentally sustainable choice for CMP slurry formulation [28]. The substrates to be polished were silicon wafers with a thermally grown SiO_2_ layer (20 × 20 mm^2^, oxide thickness 1000 nm) purchased from Kaihua Jingxin Electronics Co., Ltd. (Quzhou, China). All polishing experiments were carried out on a UNIPOL 1200S automatic pressure grinding/polishing machine (Shenyang, China) equipped with a polyurethane polishing pad, and three wafers were polished simultaneously in each run. The main polishing parameters were as follows: upper and lower plate rotation speeds of 60 rpm in opposite directions, a slurry flow rate of 33 mL/min, a polishing pressure of 3.56 psi, and a polishing time of 90 s. The polishing-related parameters are summarized in Table S1. After polishing, the silicon wafers were sequentially rinsed with deionized water and anhydrous ethanol, then immersed in anhydrous ethanol and ultrasonicated for 15 min, followed by natural drying. The surfaces before and after polishing were characterized by atomic force microscopy (AFM, Cypher S, Oxford Instruments Asylum Research, Inc., Santa Barbara, CA, USA) operated in tapping mode using an HQ:NSC18/Pt probe (Mikromasch, Watsonville, CA, USA) at a scan rate of 1 Hz over a scan area of 5.0 × 5.0 μm^2^. Three random positions were selected on each silicon wafer for AFM characterization, resulting in a total of nine locations, in order to ensure the reliability of the roughness measurement. The surface roughness parameters, Ra and Rq were directly read from the raw AFM topography data using Asylum Research software (Version 13.11.94) under the Height Retrace mode. The material removal rate (MRR) was calculated via the mass-loss method, as described in Equation (1) [29]:

(1) $$MRR=\frac{m_{0}-m}{\rho \cdot S\cdot t}$$

*m*_0_ and *m* are the masses of the wafer before and after polishing, *ρ* is the density of the oxide layer (2.2 g/cm^3^), *S* is the surface area of the wafer, and *t* is the polishing time.

## 3. Results and Discussion

### 3.1. Synthesis and Optimization of the Composite Material SiO_2_@ZrO_2_

**Figure 2:**
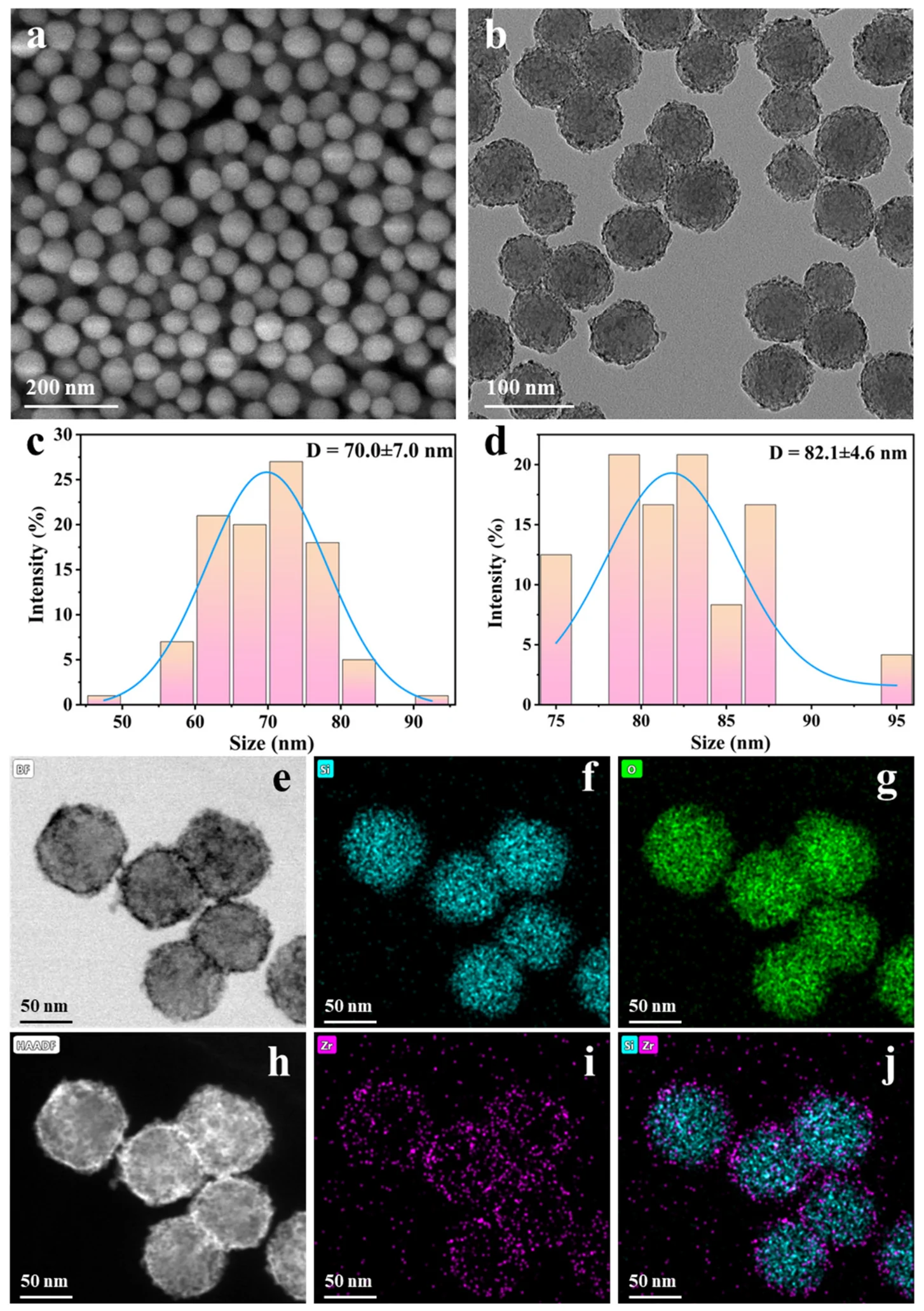
(**a**) SEM image of SiO_2_; (**b**) TEM image of SiO_2_@ZrO_2_; (**c**,**d**) particle size distribution of SiO_2_ and SiO_2_@ ZrO_2_; (**e**–**j**) HAADF-STEM image of SiO_2_@ZrO_2_ and associated EDS mapping.

The morphology of SiO_2_ was characterized by SEM. The SiO_2_ particles (Figure 2a) exhibit a uniformly spherical shape with good dispersion. TEM observation of SiO_2_@ZrO_2_ (Figure 2b) reveals a uniform morphology and favorable dispersion, which is beneficial for reducing mechanical scratches and surface defects during polishing, thereby improving the polishing quality. Compared with the SiO_2_ core before coating, the outer surface appears relatively rough, indicating that the SiO_2_ has been successfully coated to form a core–shell material. Statistical analysis of 100 particles in the SEM images using Nano Measurer yielded a SiO_2_ particle size of 70.0 ± 7.0 nm (Figure 2c), while the particle size of SiO_2_@ZrO_2_ was determined to be 82.1 ± 4.6 nm (Figure 2d). To further confirm the successful formation of the core–shell structure, HAADF-STEM imaging was performed on SiO_2_@ZrO_2_. As shown in Figure 2e,h, a strong contrast between the core and the shell can be clearly observed: the gray region corresponds to the SiO_2_ core, while the dark region in Figure 2e and the bright region in Figure 2h represent the outer ZrO_2_ shell. Additionally, EDS characterization of the composite material was carried out (Figure 2f,g,i,j). The Si-Zr elemental overlay mapping (Figure 2j) clearly demonstrates that Si is predominantly distributed in the interior region of the particle, whereas Zr is concentrated in the peripheral region. To further verify the continuity and uniformity of the ZrO_2_ shell, EDS elemental line-scanning analysis was performed on the SiO_2_@ZrO_2_ core–shell particles (Figure S1). The results reveal that the Si signal reaches its maximum at the particle center and gradually decreases toward the edge, while the Zr signal exhibits a distinct intensity peak at the peripheral region with negligible intensity in the core region. This characteristic clearly demonstrates that the ZrO_2_ shell continuously coats the SiO_2_ core, with a well-defined and sharp interface between the core and the shell. Furthermore, the statistical analysis of the 30 particles in Figure 2b revealed that the average thickness of the shell layer was 6.22 ± 0.81 nm, with a relatively narrow distribution. (Figure S2). These results further verify the successful fabrication of the SiO_2_@ZrO_2_ core–shell material. This complete shell structure ensures that each abrasive particle can fully utilize the high hardness of ZrO_2_ during the polishing process, avoiding the weakening of the rigidity constraint effect caused by incomplete shell layers due to “soft core exposure”. The uniform core–shell structure also provides a structural basis for the estimation of the effective elastic modulus in the subsequent Hertz contact analysis.

**Figure 3:**
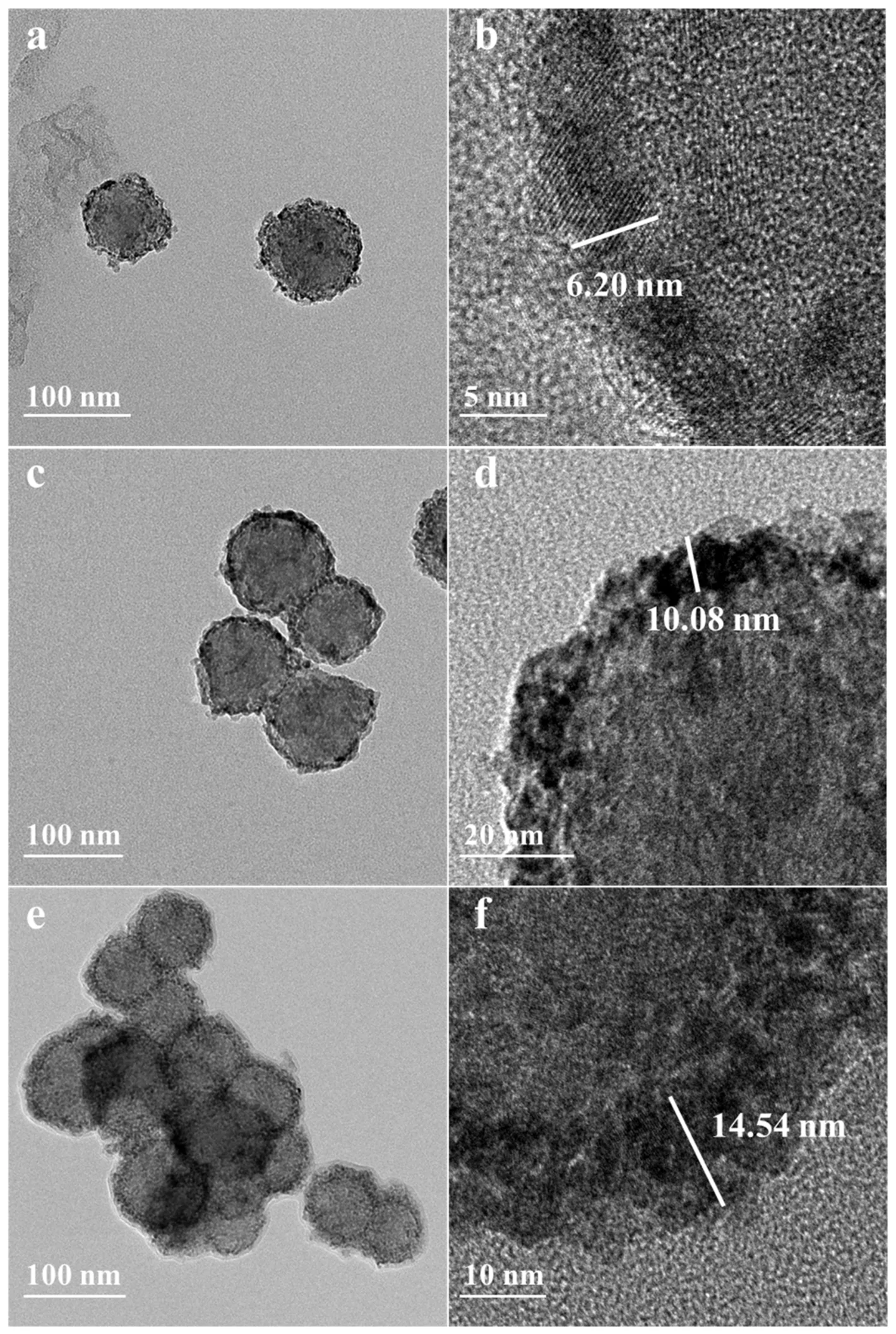
TEM images of SiO_2_@ZrO_2_ at different zirconium source concentrations: (**a**,**b**) 0.8 mL; (**c**,**d**) 1.6 mL; (**e**,**f**) 3.2 mL.

Figure 3 presents the TEM images of SiO_2_@ZrO_2_ synthesized with different concentrations of zirconium n-butoxide. When the amounts of the zirconium source were 0.8, 1.6, and 3.2 mL, the corresponding shell thicknesses were 6.20, 10.08, and 14.54 nm, respectively. This indicates that the thickness of the shell layer is positively correlated with the amount of zirconium precursor used. Hence, the thickness of the ZrO_2_ shell can be readily tuned by varying the dosage of the zirconium source. However, as the zirconium source concentration increased, the degree of particle agglomeration became more pronounced. It is possible that additional mechanical scratches may be introduced during the actual polishing process. Therefore, considering the balance between the shell layer thickness and dispersion, 0.8 mL of zirconium source was selected for the subsequent polishing experiment to achieve a unified high removal rate and low damage.

**Figure 4:**
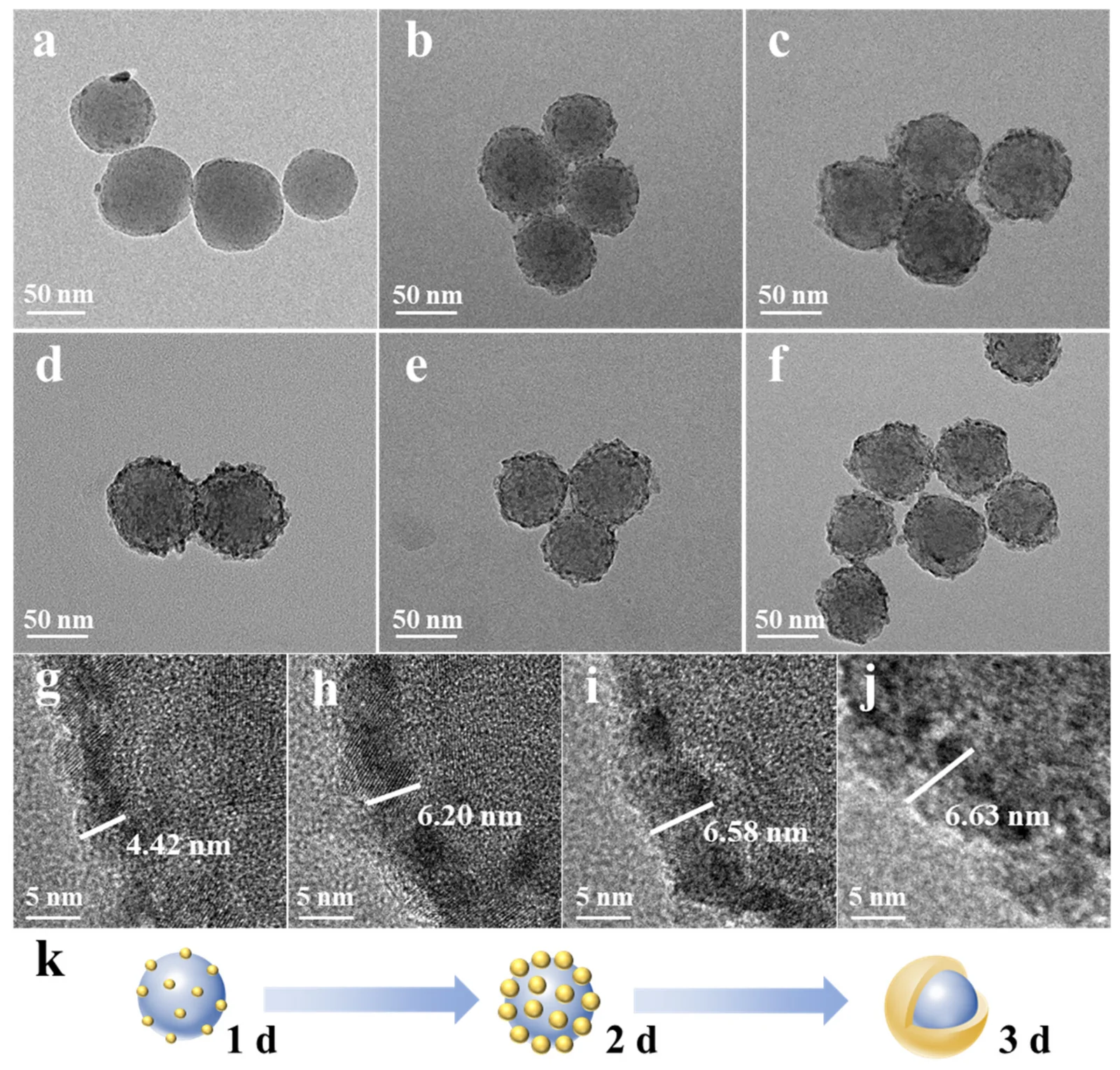
TEM images of SiO_2_@ZrO_2_ at different aging times: (**a**) 0 days; (**b**) 1 day; (**c**,**g**) 2 days; (**d**,**h**) 3 days; (**e**,**i**) 4 days; (**f**,**j**) 5 days. (**k**) Schematic diagram of the aging process.

During the synthesis of SiO_2_@ZrO_2_, the aging step is particularly critical, as it directly governs the coating integrity of the core–shell material [30]. Therefore, the effect of aging time on the product was investigated. Figure 4 shows the morphologies of the SiO_2_@ZrO_2_ core–shell material obtained at different aging times. The gray central region represents the SiO_2_ core, while the dark outer layer corresponds to the ZrO_2_ shell. It can be observed that without aging, only a small amount of ZrO_2_ particles were attached to the SiO_2_ surface (Figure 4a), and a complete coating was not achieved. With prolonged aging time, more ZrO_2_ particles adhered to the SiO_2_ surface and gradually formed an intact coating (Figure 4b–f). A schematic illustration of the shell evolution during the aging process is presented in Figure 4k. The shell thicknesses at 2, 3, 4, and 5 days of aging were 4.42, 6.20, 6.58, and 6.63 nm, respectively (Figure 4g–j). After 3 days of aging, the shell thickness showed little further change; thus, considering the time cost, and the complete shell layer can evenly distribute the polishing pressure over the entire particle, avoiding damage to the wafer surface caused by local stress concentration, the aging time was determined to be 3 days. The thickness of the shell layer under different conditions is shown in Table 1.

### 3.2. Structural Characterizations of Composite Material SiO_2_@ZrO_2_

**Figure 5:**
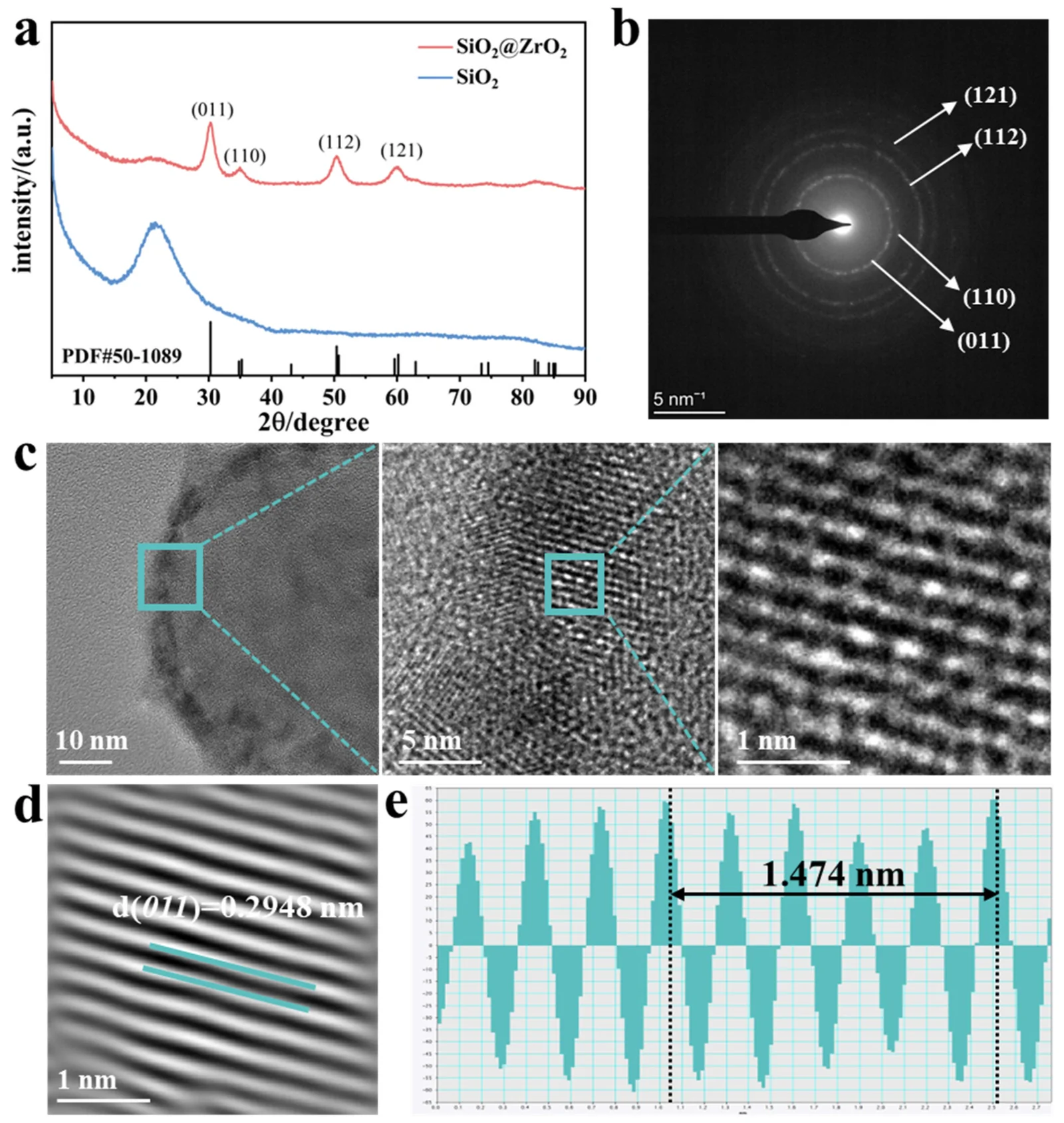
(**a**) XRD patterns of SiO_2_ and SiO_2_@ZrO_2_; Structural characterization of SiO_2_@ZrO_2_: (**b**) SAED pattern; (**c**) HRTEM image; (**d**) IFFT pattern corresponding to c; (**e**) lattice spacing distribution pattern in (**d**).

Figure 5a presents the XRD patterns of SiO_2_ and the SiO_2_@ZrO_2_ composite, both of which had been calcined at 900 °C. A prominent broad diffraction peak observed at approximately 22° is characteristic of amorphous SiO_2_ [14]; in the composite, the intensity of this peak is significantly weakened due to the coating of SiO_2_. Moreover, the diffraction peaks of SiO_2_@ZrO_2_ match well with the standard card of tetragonal ZrO_2_ (PDF#50-1089). Four major diffraction peaks are observed at 30.27°, 35.26°, 50.38°, and 60.21°, corresponding to the (011), (110), (112), and (121) crystal planes of tetragonal ZrO_2_, respectively. The XRD results of the composite indicate that a tetragonal ZrO_2_ shell with good crystallinity has been formed on the surface of the amorphous SiO_2_ core. The Raman spectrum can also prove this point (Figure S3) [S1,S2]. The crystallite size of the ZrO_2_ shell calculated using the Debye–Scherrer formula is 4.9 nm. Figure 5b shows the selected-area electron diffraction (SAED) pattern of the ZrO_2_ shell, displaying distinct diffraction rings that are indexed to the (011), (110), (112), and (121) planes. This confirms the polycrystalline nature of the ZrO_2_ shell and is consistent with the XRD results. The high-resolution transmission electron microscopy (HRTEM) image in Figure 5c clearly reveals the lattice fringes of the ZrO_2_ shell. Further inverse fast Fourier transform (IFFT) processing yields Figure 5d, and the lattice spacing measurement in Figure 5e gives a value of 0.2948 nm, which corresponds to the (011) plane of tetragonal ZrO_2_.

**Figure 6:**
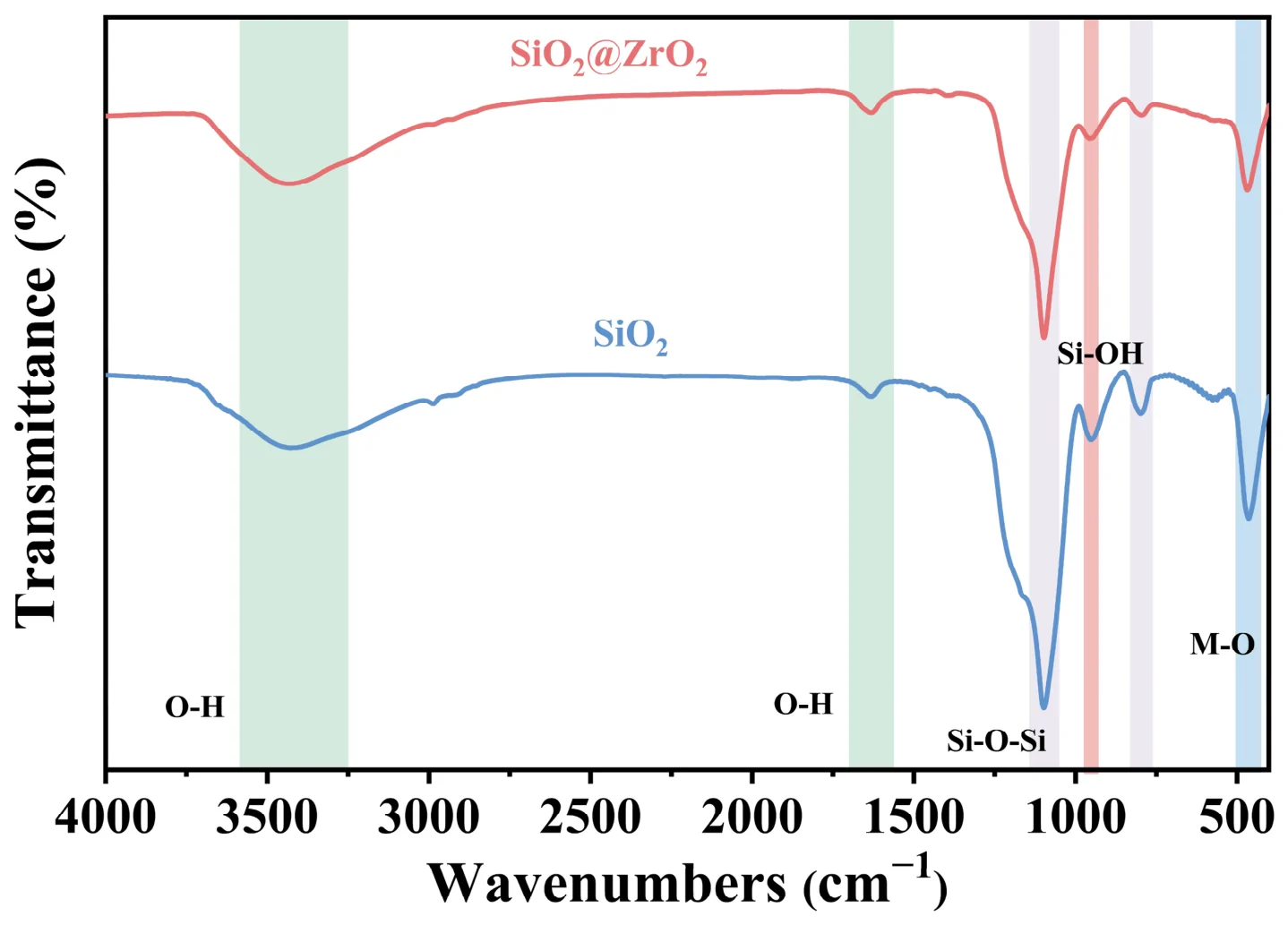
FT-IR spectra of SiO_2_ and SiO_2_@ZrO_2_.

The surface chemical compositions of SiO_2_ and SiO_2_@ZrO_2_ were analyzed by FT-IR. As shown in Figure 6, the characteristic peaks at 3430 cm^−1^ and 1633 cm^−1^ correspond to the stretching vibration and bending vibration of –OH groups from water, respectively [31]. The peaks at 1098 cm^−1^, 795 cm^−1^, and 468 cm^−1^ are attributed to the asymmetric stretching, symmetric stretching, and bending vibrations of Si–O–Si, respectively [32,33]. The persistence of these peaks in the coated SiO_2_@ZrO_2_ indicates that the original structure is preserved after coating. The peak at 954 cm^−1^ is assigned to Si–OH vibrations [34]. The characteristic peak of Zr–O–Zr at 472 cm^−1^ [35] overlaps with the bending vibration peak of Si–O–Si and is therefore not discernible. A comparison of the characteristic peaks before and after coating reveals that the intensity of the Si–OH peak decreases after coating, suggesting that a condensation reaction occurs between the hydrolyzed products of the zirconium source and Si–OH groups, thereby achieving the coating. The intensity of the Si–O–Si peaks also decreases after coating, further indicating that the SiO_2_ surface is covered by another substance and corroborating the core–shell structure. The presence of the Si–O–Zr vibrational absorption peak cannot be determined owing to its overlap with other peaks.

**Figure 7:**
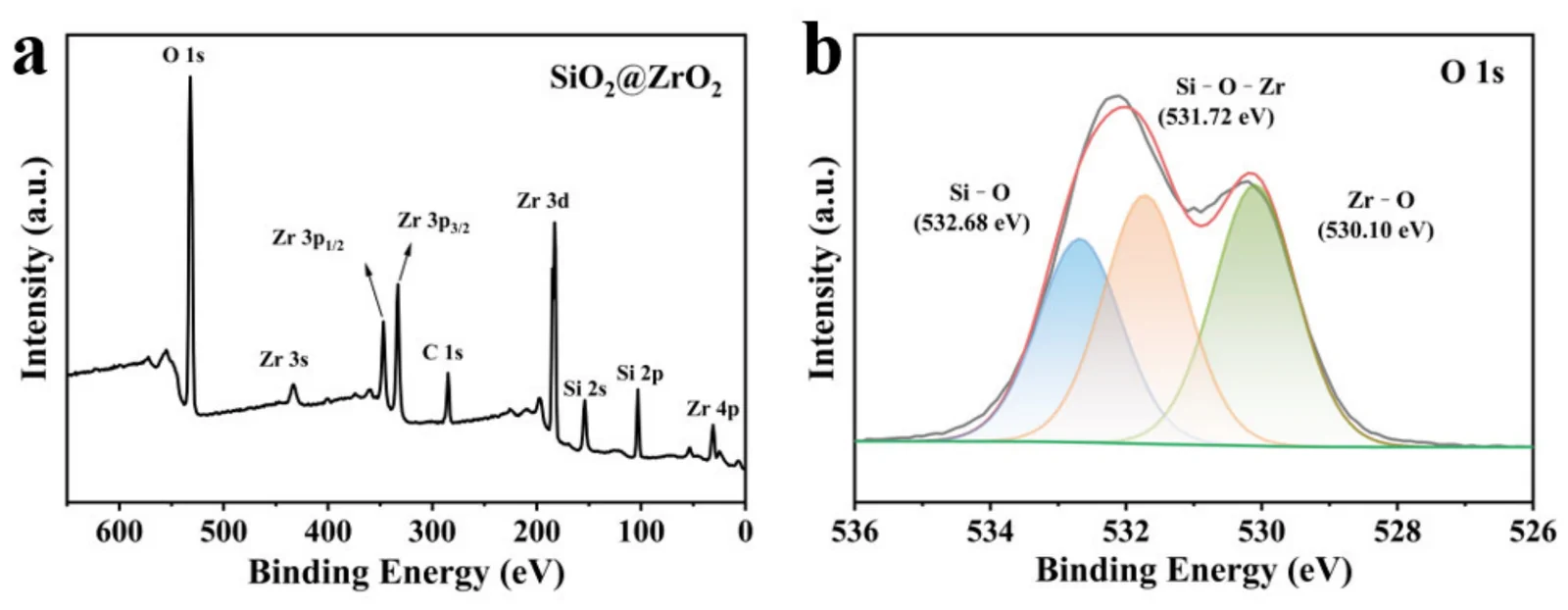
XPS spectra of SiO_2_@ZrO_2_: (**a**) full spectrum; (**b**) O 1s detailed spectrum.

To further analyze the elemental composition and chemical states of SiO_2_@ZrO_2_ and to confirm the formation of Si–O–Zr bonds, X-ray photoelectron spectroscopy (XPS) was employed for further characterization. The survey spectrum (Figure 7a) confirms the presence of Zr, Si, O, and C elements. The C 1s peak originates from surface contamination, while the Zr 3d and Si 2p peaks indicate the existence of ZrO_2_ and SiO_2_ [36]. In the high-resolution O 1s spectrum (Figure 7b), the peaks at 532.68 eV and 530.10 eV are assigned to Si–O bonds and Zr–O bonds, respectively [37]. The peak at 531.72 eV is attributed to the chemical shift caused by Si–O–Zr bonding [38]; when Si–O bonds with Zr, the O 1s binding energy decreases. Although the identification of individual XPS peaks may not be entirely conclusive, the O 1s peak at 531.72 eV, combined with the structural stability demonstrated by the core–shell structure observed by TEM, serves as a strong indication, strongly suggests that the SiO_2_ core and the ZrO_2_ shell are chemically bonded through Si–O–Zr linkages. The formation of chemical bonds can ensure the stability of the shell layer during the CMP process and prevent the shell layer from falling off.

**Figure 8:**
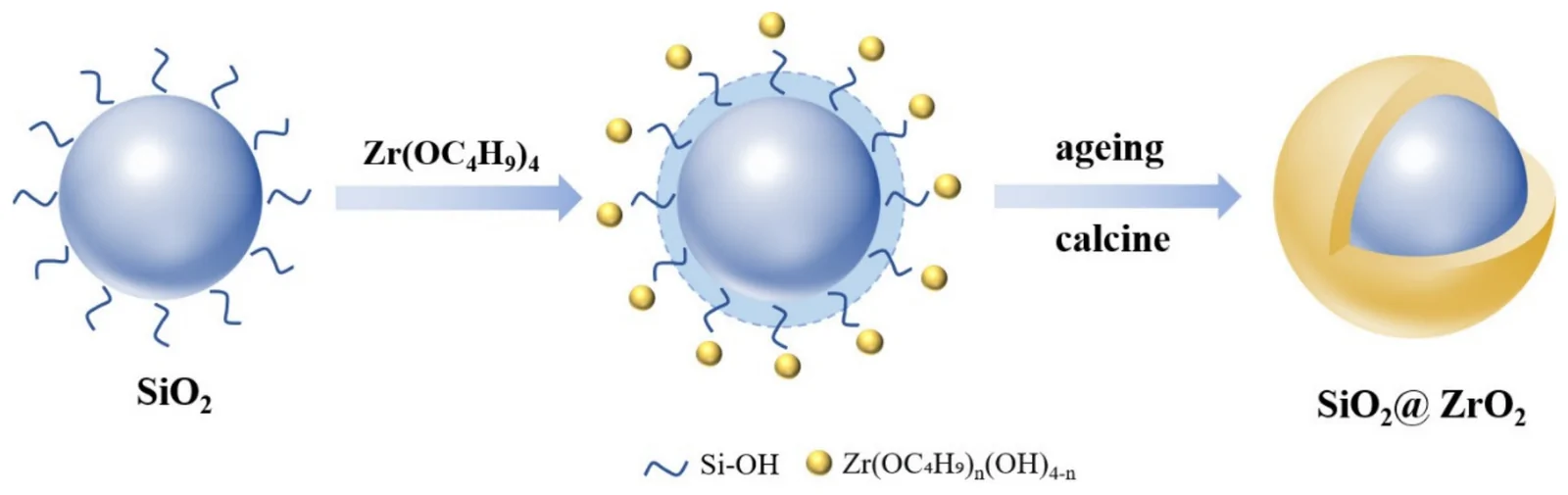
The synthesis mechanism diagram of SiO_2_@ZrO_2_.

In conclusion, we can obtain the synthesis mechanism of SiO_2_@ZrO_2_ (Figure 8). The hydrolysis of n-butyl zirconium will result in a product containing –OH, namely Zr(OC_4_H_9_)_n_(OH)_4−n_. The hydrolysis-condensation equation is as follows (Equations (2)–(6)):

Zr(OC_4_H_9_)_4_ + H_2_O → Zr(OC_4_H_9_)_3_(OH) + C_4_H_9_OH (2)

Zr(OC_4_H_9_)_3_(OH) + H_2_O → Zr(OC_4_H_9_)_2_(OH)_2_ + C_4_H_9_OH (3)

Zr(OC_4_H_9_)_2_(OH)_2_ + H_2_O → Zr(OC_4_H_9_)(OH)_3_ + C_4_H_9_OH (4)

Zr(OC_4_H_9_)(OH)_3_ + H_2_O → Zr(OH)_4_ + C_4_H_9_OH (5)

Si-OH + HO-Zr → Si-O-Zr + H_2_O (6)

The hydrolysis products of the zirconium source undergo condensation reactions with the excess –OH groups on the SiO_2_ surface to form Si–O–Zr chemical bonds [34]. Upon aging, additional chemical bonds are established, and the subsequent calcination ultimately yields a stable core–shell structure.

### 3.3. Polishing Performances

The dispersion stability of the polishing slurry was evaluated prior to polishing. Upon the addition of γ-PGA, the zeta potential decreased (i.e., became more negative) from −31.65 mV to −36.18 mV. Generally, a zeta potential with an absolute value exceeding 30 mV indicates sufficient electrostatic repulsion to maintain colloidal stability [39]. The observed increase in the absolute zeta potential upon γ-PGA addition confirms that the adsorbed γ-PGA chains impart a higher negative charge density to the particle surfaces, thereby strengthening the electrostatic repulsive forces between neighboring particles and effectively counteracting the van der Waals attractive forces that otherwise induce agglomeration.

Interestingly, the hydrodynamic diameter measured by DLS increased substantially from 258.13 nm to 2084.73 nm upon γ-PGA addition. This increase, rather than indicating particle agglomeration, is a direct consequence of the formation of a polymer adsorption layer on the ZrO_2_ particle surfaces. DLS measures the hydrodynamic diameter, which includes not only the inorganic particle core but also the solvation layer and the adsorbed polymer layer. γ-PGA is a high-molecular-weight anionic polyamino acid with flexible chains that adopt an extended conformation in aqueous solution. Upon adsorption onto the ZrO_2_ particles via electrostatic or coordination interactions, the polymer chains form a dense polymeric brush layer on the particle surface, significantly increasing the hydrodynamic radius of the particle–polymer complex and leading to the observed eightfold increase in apparent particle size. This substantial increase provides compelling evidence for the successful adsorption of γ-PGA onto the ZrO_2_ particles. The adsorbed polymer layer provides additional steric hindrance, which, together with the enhanced electrostatic repulsion (as evidenced by the increased zeta potential), confers a dual stabilization mechanism—namely, electrosteric stabilization—to the ZrO_2_ suspension. This synergistic effect ensures excellent dispersion stability and prevents particle agglomeration during subsequent storage and CMP applications.

**Figure 9:**
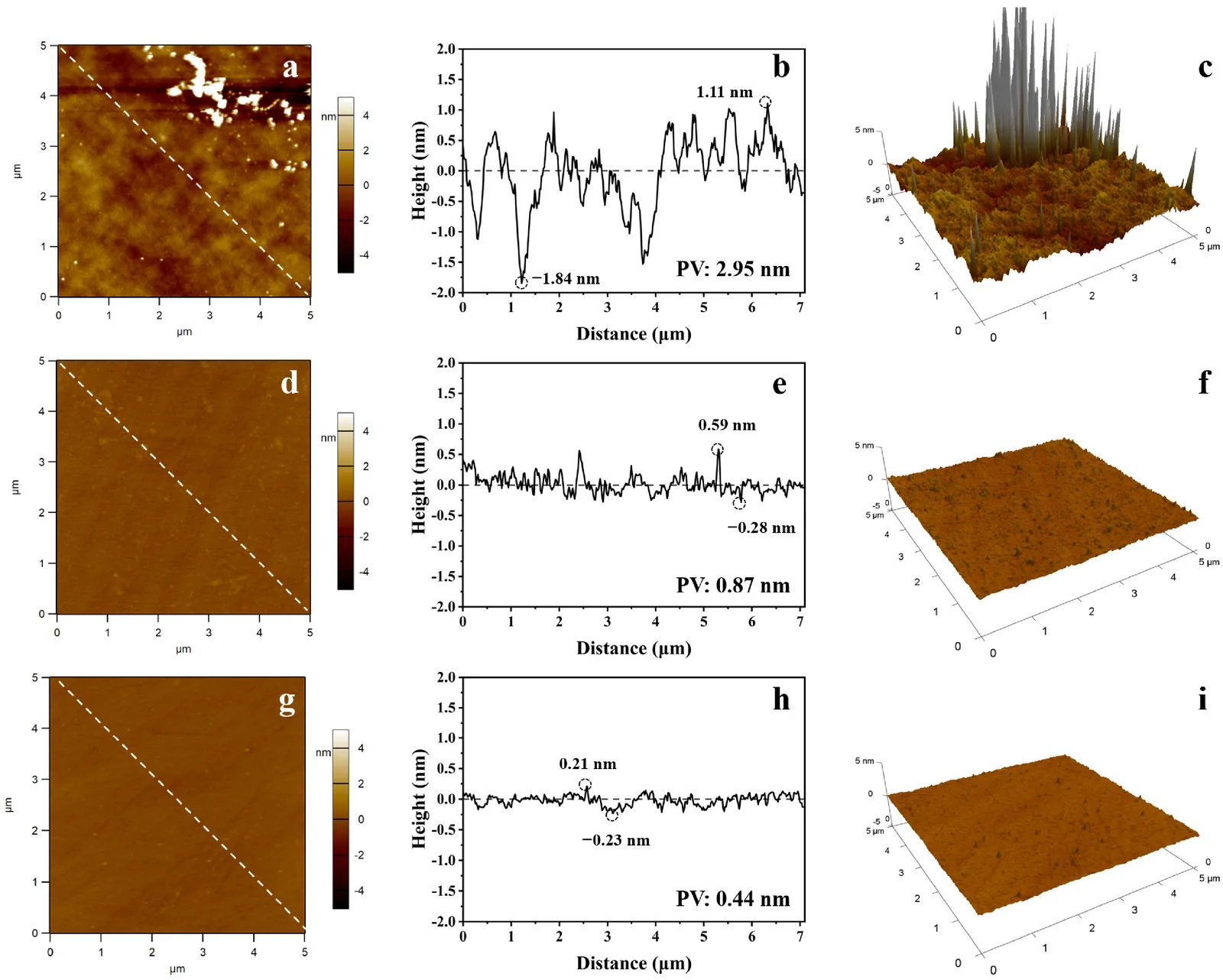
AFM surface morphology: (**a**) unpolished; (**d**) SiO_2_; (**g**) SiO_2_@ZrO_2_. Height distribution curve: (**b**) unpolished; (**e**) SiO_2_; (**h**) SiO_2_@ZrO_2_. AFM three-dimensional image: (**c**) unpolished; (**f**) SiO_2_; (**i**) SiO_2_@ZrO_2_. The images shown are representative, selected from one randomly chosen scan area on one of the three wafers per condition.

Surface roughness is a critical parameter for evaluating polishing performance, with lower roughness indicating superior polishing quality. In this work, atomic force microscopy (AFM) was employed to acquire topographical images and specific roughness data of the silicon wafer surfaces before and after polishing. Figure 9 displays representative AFM topography images of the wafer surfaces before polishing and after polishing with SiO_2_ and SiO_2_@ZrO_2_ abrasives (one randomly selected scan area from one of the three wafers per condition). For the unpolished wafer, the two-dimensional AFM image (Figure 9a) clearly reveals uneven color contrast, inhomogeneous distribution, and the presence of white impurities. The measured arithmetic average roughness (Ra) and root-mean-square roughness (Rq) are 0.871 nm and 1.860 nm, respectively, indicating a relatively rough surface. A line scan along the white dashed line in the 2D image yields the height profile (Figure 9b), showing a maximum peak height of 1.11 nm and a maximum valley depth of 1.84 nm, resulting in a peak-to-valley (PV) value of 2.95 nm. The corresponding three-dimensional AFM image (Figure 9c) intuitively displays the pronounced undulations and poor flatness of the unpolished surface. After polishing with SiO_2_ and SiO_2_@ZrO_2_ particles, the 2D AFM images (Figure 9d,g) exhibit significantly improved color uniformity compared to the unpolished surface. The measured average surface roughness values are as follows: for SiO_2_, Ra = 0.173 ± 0.037 nm, Rq = 0.334 ± 0.151 nm; for SiO_2_@ZrO_2_, Ra = 0.105 ± 0.015 nm, Rq = 0.138 ± 0.024 nm, demonstrating a marked reduction in surface roughness after polishing. The detailed roughness data from all nine AFM measurement points are provided in Table S2. Notably, the core–shell structured SiO_2_@ZrO_2_ material achieves an even better roughness reduction, reaching near atomic-scale polishing and delivering outstanding polishing performance. A two-tailed *t*-test confirmed that the difference between the two groups is statistically significant (*p* < 0.05). For the SiO_2_-polished surface, the maximum peak height is 0.59 nm, the maximum valley depth is 0.28 nm, and the PV value is 0.87 nm (Figure 9e). In contrast, the SiO_2_@ZrO_2_-polished surface exhibits a maximum peak height of 0.21 nm, a maximum valley depth of 0.23 nm, and a PV value of 0.44 nm (Figure 9h). The corresponding 3D images (Figure 9f,i) confirm that the surface undulations after both polishing treatments are substantially smaller than those of the unpolished wafer, with the SiO_2_@ZrO_2_-polished surface displaying the least undulation. In summary, the surface roughness follows the order: SiO_2_@ZrO_2_ < SiO_2_. This can be attributed to the difference in hardness between the two materials. The hardness of SiO_2_ is lower than that of ZrO_2_; therefore, the SiO_2_@ZrO_2_ abrasive with a harder outer shell is less prone to elastic deformation and can maintain its spherical morphology. This prevents the transition from rolling friction to sliding friction caused by deformation, thereby avoiding sliding plowing [40] and yielding a lower surface roughness. Furthermore, under the same polishing pressure, the indentation depth of the abrasive into the workpiece surface is inversely proportional to the abrasive hardness [24,41]. Consequently, compared with pure SiO_2_ abrasive, the SiO_2_@ZrO_2_ abrasive produces a shallower indentation depth on the workpiece, leading to finer surface scratches and lower surface roughness.

**Figure 10:**
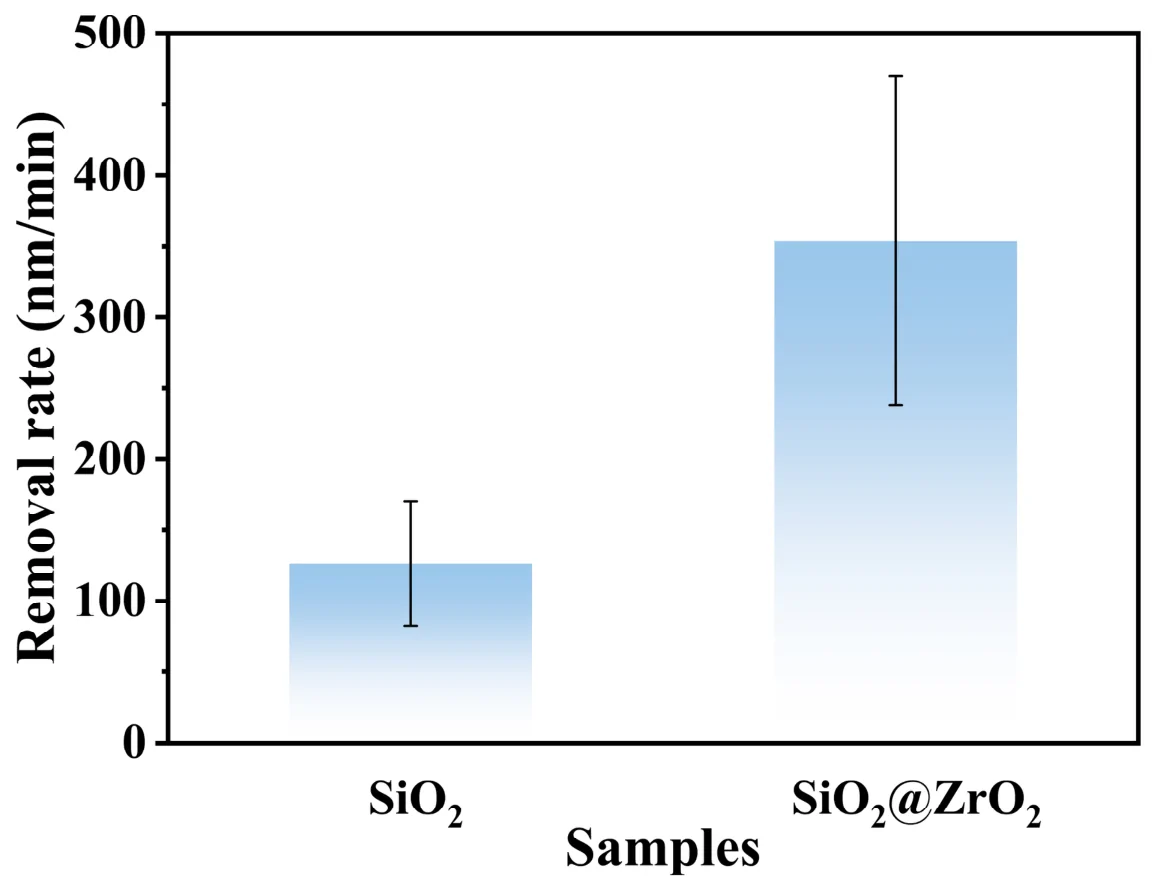
Material removal rates of SiO_2_ and SiO_2_@ZrO_2_ abrasives.

MRR is another key parameter for evaluating polishing performance, as it quantifies the polishing speed. Figure 10 presents the MRR values obtained with different abrasives. As shown, the MRR of the SiO_2_@ZrO_2_ core–shell abrasive was determined to be 353.54 ± 115.73 nm/min, which is approximately 2.8 times that of pure SiO_2_ abrasives (126.26 ± 43.73 nm/min), demonstrating the superior polishing efficiency of the core–shell abrasive. The core–shell abrasive achieves a significantly lower surface roughness compared to pure SiO_2_ (the reduction in Ra is approximately 39%), confirming its superior surface planarization capability. The difference in roughness between the two groups is statistically significant (*p* < 0.05). The relatively large standard deviation is primarily attributed to the short polishing duration (90 s) and the correspondingly small mass loss during each run, which amplifies the relative measurement uncertainty. However, multiple MRR results (Table S3) all indicate that the MRR of the core–shell material SiO_2_@ZrO_2_ is significantly greater than that of pure SiO_2_.

It is worth noting that the same mass fraction (0.5 wt%) was used for both SiO_2_ and SiO_2_@ZrO_2_ abrasives in this study. However, the density of ZrO_2_ is greater than that of SiO_2_, the particle number concentration of the SiO_2_@ZrO_2_ abrasive in the slurry is significantly lower than that of pure SiO_2_ at the same mass fraction. This means that, if any, the SiO_2_@ZrO_2_ slurry contains fewer abrasive particles per unit volume, which would tend to reduce the number of active contact sites during polishing. Nevertheless, despite this lower particle number concentration, the SiO_2_@ZrO_2_ abrasives still achieve a substantially higher MRR (353.5 nm/min) than pure SiO_2_ (126.3 nm/min). This further confirms that the performance enhancement originates primarily from the superior mechanical properties of the ZrO_2_ shell rather than from a higher particle concentration.

We have compared the polishing performance obtained in this study with that of other abrasives, including SiO_2_-based abrasives as well as CeO_2_ and Al_2_O_3_ abrasives, as summarized in Table 2. It can be observed that our SiO_2_@ZrO_2_ abrasive offers a competitive MRR while achieving near-atomic-scale surface roughness, representing a favorable balance between removal rate and surface quality compared to other SiO_2_-based composite systems reported in the literature.

### 3.4. Mechanical Analysis Based on Hertz Contact Theory

To gain a mechanistic understanding of the role of the rigid ZrO_2_ shell in modulating the contact behavior during CMP, we analyzed the elastic contact between a single spherical abrasive particle and the wafer surface based on classical Hertzian contact theory [45]. In the CMP process, the applied pressure is transmitted through the polishing pad and distributed among the abrasive particles trapped at the pad–wafer interface. For a spherical particle of radius R in elastic contact with a flat surface under a normal load P, Hertzian theory gives the contact radius a and indentation depth δ as follows:

(7) $${a=\left(\frac{3\mathrm{PR}}{4E_{r}}\right)}^{1/3}$$

(8) $$\delta =\frac{a^{2}}{R}={\left(\frac{9P^{2}}{16RE_{r}}\right)}^{1/3}$$

E_r_ is the reduced elastic modulus of the abrasive–wafer contact system, defined as:

(9) $$\frac{1}{E_{r}}\;=\;\frac{1{-v}_{p}^{2}}{E_{p}}\;+\;\frac{1{-v}_{w}^{2}}{E_{w}}$$

E_p_ and V_p_ are the elastic modulus and Poisson’s ratio of the abrasive particle, respectively. E_w_ and V_w_ are the elastic modulus and Poisson’s ratio of the wafer, respectively. From Equations (7) and (8), it can be seen that both a and δ decrease with increasing Er.

For the core–shell particle, the effective elastic modulus E_eff_ is governed by its composite structure. This effective modulus can be approximated as follows:

(10) $$E_{\mathrm{eff}}\;=\;E_{\mathrm{shell}}\cdot V_{\mathrm{shell}}\;+\;E_{\mathrm{core}}\cdot V_{\mathrm{core}}$$

Here, V_shell_ and V_core_ represent the volume fractions of the shell layer and the core respectively.

According to the literature, the elastic moduli of SiO_2_ and ZrO_2_ are 69 and 210 GPa [46,47], and their Poisson’s ratios are 0.17 and 0.31 [48,49], respectively. Based on these parameters, the effective elastic modulus of the core–shell particle is estimated to be 122 GPa, substantially exceeding that of bare SiO_2_. Consequently, under identical loading conditions, the core–shell abrasive is expected to yield a reduced contact area and a shallower indentation depth.

Specifically, a smaller contact area implies a higher local shear stress at the abrasive–wafer interface under the same total load. This increase in shear stress directly facilitates material removal, which is consistent with the experimentally observed enhancement in MRR from 126.26 nm/min (for pure SiO_2_) to 353.54 nm/min (for SiO_2_@ZrO_2_). Meanwhile, the shallower indentation depth effectively suppresses the “plowing effect,” minimizing the generation of scratches and subsurface damage. This directly accounts for the superior surface quality achieved with the SiO_2_@ZrO_2_ abrasive compared to pure SiO_2_.

In summary, the qualitative analysis based on Hertzian contact theory validates the proposed “rigid constraint” mechanism: the high-modulus ZrO_2_ shell increases the effective elastic modulus of the composite abrasive, reducing the contact area to concentrate shear stress for enhanced MRR, while simultaneously limiting the indentation depth to mitigate mechanical damage and achieve superior surface quality.

### 3.5. Removal Mechanism

**Figure 11:**
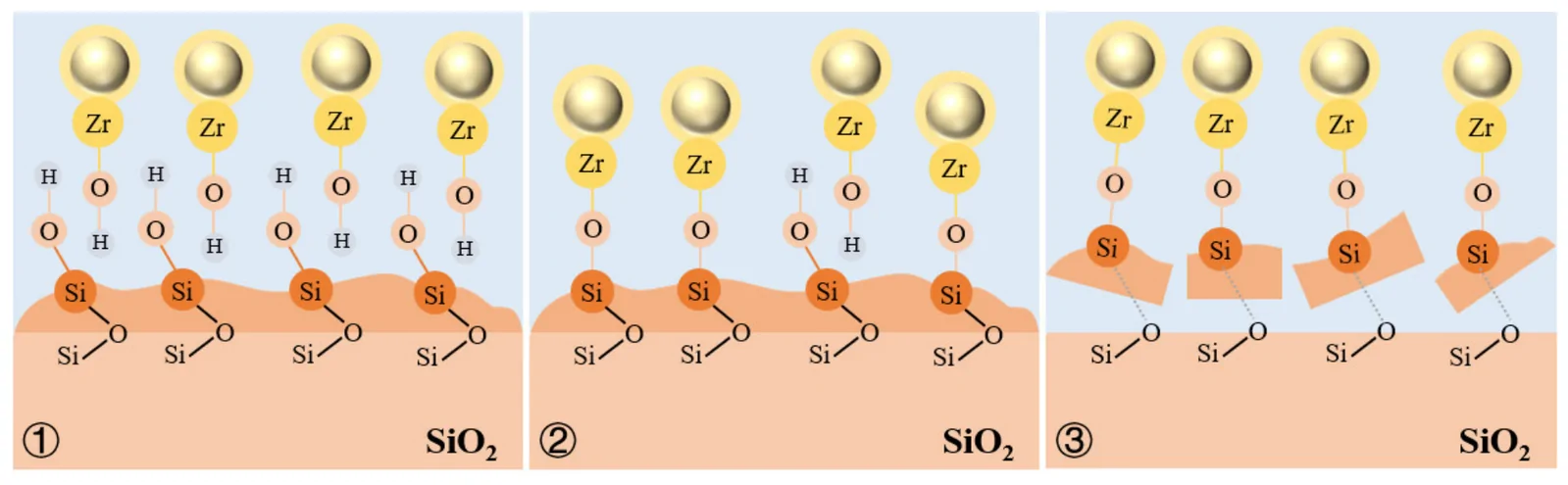
Material removal mechanism diagram.

It is generally accepted that chemical interactions dominate the material removal process during CMP [50,51]. According to the “chemical tooth” theory proposed by Cook [52], metal oxide abrasives can form new M–O–Si bonds (M = Ce, Zr) with the silicon wafer surface after polishing, thereby facilitating material removal. To reveal the polishing mechanism more intuitively, Gao et al. [27] simulated the reaction process between CeO_2_ abrasives and the silicon wafer surface by studying the adsorption behavior of CeO_2_ clusters on Si/SiO_2_ surfaces and found the formation of Ce–O–Si bonds, which corroborates the mechanism proposed by Cook. Furthermore, Li [53] confirmed the generation of Ce–O–Si bonds during CMP through a combination of XPS characterization and molecular dynamics simulations. Regarding the polishing mechanism of ZrO_2_, Dai et al. [23] polished ZrO_2_ ceramics with SiO_2_ abrasives and experimentally demonstrated the formation of Zr–O–Si bonds. Based on the above, it is reasonable to infer that in the present work, Zr-O-Si bonds are formed when the SiO_2_@ZrO_2_ composite abrasive polishes SiO_2_, thereby achieving effective material removal from the silicon wafer (Figure 11).

## 4. Conclusions

Addressing the long-standing challenge in silicon wafer chemical mechanical polishing of simultaneously achieving a high material removal rate and atomic-scale surface quality, this study proposes a novel design strategy for SiO_2_@ZrO_2_ core–shell abrasives based on a rigid ZrO_2_ shell. By regulating the precursor ratio and aging time during the sol–gel process, the controllable synthesis of sub-100 nm composite abrasives was successfully realized. Systematic characterization strongly indicates that the tetragonal ZrO_2_ shell is firmly anchored to the surface of the SiO_2_ core through Si–O–Zr chemical bonds, endowing the abrasive with excellent structural stability.

The polishing experimental results demonstrate that the SiO_2_@ZrO_2_ abrasive breaks through the limitations of conventional abrasive design, achieving a simultaneous leap forward in both polishing efficiency and surface quality. Benefiting from the high hardness of the ZrO_2_ shell, the abrasive effectively resists elastic deformation under polishing pressure, not only raising the material removal rate to 353.54 ± 115.73 nm/min but also, for the first time, reducing the surface roughness to Ra = 0.105 ± 0.015 nm, which approaches the atomic-scale planarization standard (Ra < 0.1 nm) and represents a 40% improvement over pure SiO_2_ abrasives (Ra = 0.173 ± 0.037 nm). Horizontal comparison with literature data confirms the leading position of this abrasive among SiO_2_-based composite systems. Based on the Hertz contact theory, a mechanical analysis reveals that the rigid shell enables highly efficient chemo-mechanical synergistic removal through the dual actions of “reducing contact area to enhance shear stress” and “limiting indentation depth to mitigate mechanical damage”.

This work not only provides a highly promising high-performance polishing slurry solution for advanced semiconductor manufacturing, but also offers new theoretical perspectives for understanding the influence of abrasive mechanical properties on the CMP process. However, several limitations should be acknowledged. First, the direct experimental verification of Zr–O–Si bond formation during the actual CMP process remains challenging. Second, the current study is limited to short-duration polishing, which, although consistent with typical testing conditions reported in the literature for initial performance evaluation, does not fully reflect the demands of practical industrial applications. These aspects will be addressed in our future research. Beyond these considerations, future work will also include atomic-scale simulations of the interfacial reactions between ZrO_2_ and SiO_2_, and will investigate the applicability of the rigid core–shell structure to the polishing of large-size silicon wafers as well as third-generation semiconductors such as SiC and GaN.

## Figures and Tables

**Table 1:** The shell thickness of SiO_2_@ZrO_2_ under different conditions.

Aging Time (d)	Zirconium Precursor (mL)	Thickness (nm)
2	0.8	4.42
3	0.8	6.20
3	1.6	10.08
3	3.2	14.54
4	0.8	6.58
5	0.8	6.63

**Table 2:** Summary of polishing performance of composite abrasives.

Abrasive Types	Particle Size (nm)	Polishing Pressure and Time	Surface Roughness (nm)		MRR (nm/min)	Ref.
			Ra	Rq		
Before CMP			0.871	1.860	—	This work
SiO_2_	70	3.56 psi 90 s	0.173	0.334	126.26	This work
SiO_2_@ZrO_2_	82	3.56 psi 90 s	0.105	0.138	353.54	This work
SiO_2_@CeO_2_	150–200	3.6 psi 60 s	—	0.428	454.6	[14]
mSiO_2_@CeO_2_	250	3.3 psi 120 s	0.18	—	38	[19]
mSiO_2_@CeO_2_	320–340	22.8 kPa 60 s	—	0.20	64	[20]
DSNs-Ce	73	3.0 psi 120 s	0.141	0.182	131	[29]
DSNs-Zr	76	3.0 psi 120 s	0.118	0.149	164	[29]
DSNs-Zr/Ce	77	3.0 psi 120 s	0.110	0.145	208	[29]
CeO_2_	60	3.0 psi 60 s	0.533	—	414.1	[42]
CeO_2_	200–300	3.5 kg 180 s	0.293	0.391	337.6	[43,44]
Al_2_O_3_	100	5 kg 1 h	0.282	—	174.18	[45]

## Data Availability

The original contributions presented in this study are included in the article. Further inquiries can be directed to the corresponding author.

## Supplementary Materials

The following supporting information can be downloaded at: https://www.mdpi.com/article/10.3390/nano16171064/s1, Table S1: Polishing process parameters; Table S2: Summary of Surface Roughness; Table S3: Summary of MRR; Figure S1: (a) HAADF- STEM image of SiO_2_@ZrO_2_; (b) Corresponding EDS elemental line-scan profiles of O, Si and Zr along the black line indicated in a; Figure S2: The thickness distribution map of the SiO_2_@ZrO_2_ shell layer in the Figure 2b; Figure S3: Raman spectrum of SiO_2_@ZrO_2_. References [54,55] are cited in the Supplementary Materials.
