# Supplementary material for: High Removal Rates and Atomically Smooth Surfaces Are Achieved on Silicon Wafers Using the New SiO2@ZrO2 Core–Shell Abrasive
Source: Nanomaterials (Basel). 2026 Aug 26;16(17):1064. doi: 10.3390/nano16171064 (PMC13567100; doi:10.3390/nano16171064)
Supplement: Supplementary file 1 [file nanomaterials-16-01064-s001.zip › nanomaterials-4512029-supplementary.pdf]

Supplementary Materials

High removal rates and atomically smooth surfaces are achieved on silicon wafers using the new SiO_2@ZrO_2 core-shell abrasive

Maokui Wang [1], Weilong He [1], Kai Feng [1,2], Rui Ye [1,2], Yunci Wang [1], Weiyi Guan [1], Facheng Qiu [3], Xing Fan [1], and Renlong Liu [1,*]

1 College of Chemistry and Chemical Engineering, Chongqing University, Chongqing ,400044, China

2 Hubei Sinophorus Electronic Materials Co., Ltd., Yichang 443007, China

3 College of Chemistry and Chemical Engineering, Chongqing University of Technology, Chongqing 400054, China

* Correspondence: lrl@cqu.edu.cn

Tables

Table S1 Polishing process parameters

Upper rotational speed	60 rpm	pH	8
Lower rotational speed	60 rpm	Down pressure	3.56 psi
Slurry flow rate	33 mL/min	Polishing time	90 s

Table S2 Summary of Surface Roughness

**Abrasives**	**Sample**	**Ra (nm)**	**Rq (nm)**
**SiO_2**	1	0.134	0.213
		0.108	0.142
		0.206	0.276
	2	0.150	0.261
		0.187	0.376
		0.221	0.593
	3	0.157	0.225
		0.195	0.393
		0.195	0.529
	**Mean**	**0.173**	**0.334**
	**SD**	**0.037**	**0.151**
**SiO_2@ZrO_2**	1	0.113	0.183
		0.075	0.097
		0.110	0.141
	2	0.112	0.142
		0.118	0.149
		0.113	0.144
	3	0.117	0.147
		0.095	0.121
		0.092	0.118
	**Mean**	**0.105**	**0.138**
	**SD**	**0.015**	**0.024**

Table S3 Summary of MRR

Abrasives	Sample	Quality before CMP (g)	Quality after CMP (g)	MRR (nm/min)
**SiO_2**	1	0.4816	0.4814	151.51
	2	0.4811	0.4809	151.51
	3	0.4804	0.4803	75.76
	**Mean**			**126.26**
	**SD**			**43.73**
**SiO_2@ZrO_2**	1	0.4830	0.4825	378.79
	2	0.4833	0.4830	227.27
	3	0.4817	0.4811	454.55
	**Mean**			**353.54**
	**SD**			**115.73**

Figures

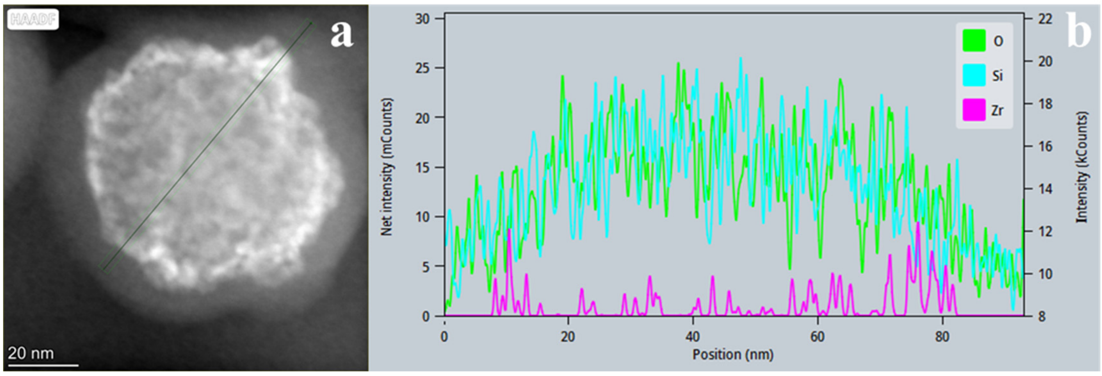

Figure S1. (a) HAADF- STEM image of $SiO_2@ZrO_2$; (b) Corresponding EDS elemental line-scan profiles of O、Si and Zr along the black line indicated in a.

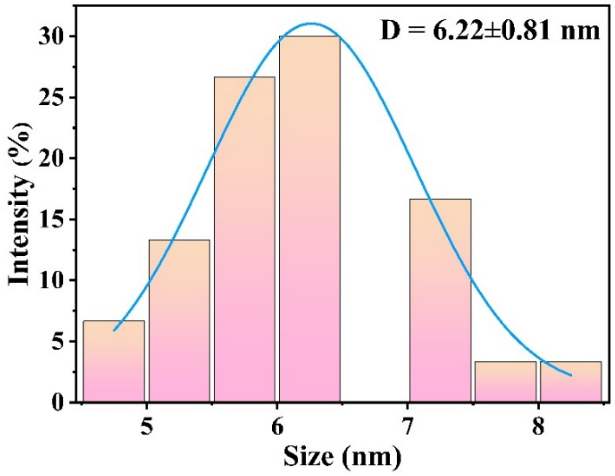

Figure S2. The thickness distribution map of the $SiO_2@ZrO_2$ shell layer in the figure2b.

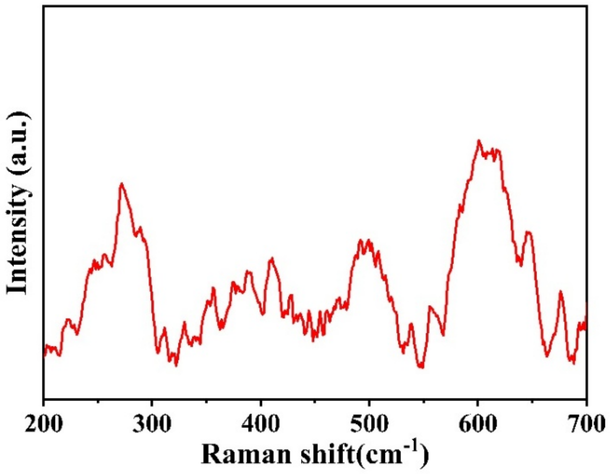

Figure S3. Raman spectrum of $SiO_2@ZrO_2$.

Raman spectrum of the $SiO_2@ZrO_2$ core-shell composite particles (Figure S3) exhibits

characteristic peaks at 272, 409, 490, 601 and 644 cm^{-1}, which are in good agreement with the reported Raman bands of tetragonal ZrO_2[1,2]. Prove that tetragonal zirconium oxide is the main crystal form.

[1] Bhaskar, S.; Awin, E.W.; Kumar, K.C.H.; Lale, A.; Bernard, S.; Kumar, R. Design of nanoscaled heterojunctions in precursor-derived t-ZrO_2/SiOC(N) nanocomposites: Transgressing the boundaries of catalytic activity from UV to visible light. *Scientific Reports* **2020**, *10*, 430. https://doi.org/10.1038/s41598-019-57394-8

[2] Basahel, S.N.; Ali, T.T.; Mokhtar, M.; Narasimharao, K. Influence of crystal structure of nanosized ZrO_2 on photocatalytic degradation of methyl orange. *Nanoscale Research Letters* **2015**, *10*, 73. https://doi.org/10.1186/s11671-015-0780-z
